# Physical analysis of the environmental impacts of fishery complementary photovoltaic power plant

**DOI:** 10.1007/s11356-022-18930-8

**Published:** 2022-02-14

**Authors:** Peidu Li, Xiaoqing Gao, Zhenchao Li, Xiyin Zhou

**Affiliations:** 1 Key Laboratory of Land Surface Process and Climate Change in Cold and Arid Regions, Northwest Institute of Eco-Environment and Resources, Chinese Academy of Sciences, Lanzhou, 730000 China; 2 College of Resources and Environment, University of Chinese Academy of Sciences, Beijing, 100049 China

**Keywords:** Fishery complementary photovoltaic power plant, Albedo, Physical model, Environmental impact

## Abstract

Photovoltaic (PV) power plants have shown rapid development in the renewable sector, but the research areas have mainly included land installations, and the study of fishery complementary photovoltaic (FPV) power plants has been comparatively less. Moreover, the mechanism of local microclimate changes caused by FPV panels has not been reported. This work revealed this mechanism using a physical model to illustrate the impact of FPV power plants in a lake on the environment. The results indicated that the lake becomes a heat sink after deploying the PV panel on water. The comprehensive albedo (0.082) decreased by 18.8% relative to the free water surface (0.101). The water energy change was dominated by the water–air vapor pressure deficit. In addition, the FPV panels had a heating effect on the ambient environment; however, the range of this effect was related to the water depth. The installation had an obvious heating effect on surface water.

## Introduction

Solar photovoltaic (PV) is the most potential renewable energy (Choi et al. 2020; Pogson et al. 2013). In recent years, the number of large-scale PV installations has shown an exponential growth trend (Barron-Gafford et al. 2016), which is likely to continue (Armstrong et al. 2016). During the period from 2009 to 2035, the predicted demand for the world’s major energies will increase by 40%, while the contribution of wind and solar energy will reach 600% (Armstrong et al. 2014). It is estimated that solar energy will meet 20–29% of global electricity demand (32,700 GW–133,000 GW) until 2100 (Breyer et al. 2017). Solar PV power generation can effectively avoid problems such as environmental pollution caused by the burning and consumption of traditional fossil energy oil, natural gas, and coal (Nugent & Sovacool 2014). Additionally, solar PV plays an important role in the promotion of zero-carbon power generation technology among international, national, and government actors to mitigate climate change (Craig et al. 2019).

With the rapid development of solar PV, the impact of large-scale deployment of PV facilities on the climate and environment has also aroused the interest and widespread concern of scholars (Hassanpour Adeh et al. 2018). During solar PV power plant construction preparation, native vegetation is removed and destroyed, and there are changes to the ground surface, such as ground fill and compaction (Hernandez et al. 2014). These changes impact the physical, chemical, and biological properties of the soil, and then affect the dynamic changes in water and nutrients, and finally, the soil serves as a medium to express vegetation and related ecological processes again. Therefore, it is important to reduce the impact of solar installation and deployment on ecological vegetation and landscape functions (Armstrong et al. 2016; Hernandez et al. 2014, 2015; Phillips 2013; Turney & Fthenakis 2011; Walston et al. 2016). There are also some studies on the impact of solar infrastructure on the environment, which focus on runoff simulation and monitoring of micro-meteorological elements (Cook 2011; Marrou et al. 2013). For example, studies have found that the removal of vegetation during the preparation stage of a PV array site degrades the soil, which leads to a significant increase in site runoff and soil erosion (Cook 2011). However, some studies have also shown that PV infrastructure is conducive to maintaining soil moisture and improving the water use efficiency of biomass and plants (Barron-Gafford et al. 2019; Hassanpour Adeh et al. 2018; Marrou et al. 2013). In addition, there is an increase in the air temperature above the PV array compared to the surrounding natural area due to the change in land-use type, vegetation coverage, and albedo (Barron-Gafford et al. 2016). Barron-Gafford et al.’s (2016) study showed that large-scale PV power plants could cause the heat island effect, and the temperature over the solar PV array increased by 3–4 °C compared with the wildland at night. However, Armstrong et al. (2016) studied the temperature changes in different areas of the PV array. The daily minimum PV array temperature was 2.4 °C higher than other areas for 1 year, and the daily maximum temperature was 6.0 °C lower than other areas. Millstein and Menon (2011) used the Weather Research and Forecasting (WRF) model to simulate the regional climate change after PV arrays were installed on roofs and pavements in the USA, and the results showed that the temperature dropped by 0.11–0.53 °C. In urban areas and at global scales, studies have shown that solar PV panels can increase the temperature of urban areas and the world by 1–2.5 °C (Hu et al. 2016). PV power plants not only affect the microclimate, but also affect the carbon cycle and biodiversity, and change the physical and chemical properties of the soil, leading to soil erosion (Armstrong et al. 2014; Hernandez et al. 2019). Additionally, studies on air quality and the energy balance of ecosystems have shown impacts, sometimes on a regional scale (Barron-Gafford et al. 2019). However, the research areas of those studies focus on land. There are some studies on water surface PV power plants. Compared with land surface PV power plants, the installation of water surface PV power plants currently focuses more on technical and economic issues. The impact of water surface PV power plants on the environment has not attracted enough attention relative to land surface PV power plants. The environmental research factors are relatively unique, and the main research is focused on the impact of water surface PV power plant on evaporation. Therefore, some scholars have noted that further study and evaluation of the impact of fishery complementary photovoltaic (FPV) facilities on the environment is warranted (Grippo et al. 2015). Although water surface PV power plants are not like land surface PV power plants that can cause water and soil loss and other environmental problems, fixed facilities such as anchor hooks and other floating bodies can cause water turbidity due to water surface fluctuations, which affects water quality. Furthermore, FPV facilities will block sunlight from passing through water bodies. Sunlight is very important for the algae that photosynthesize in the water body. In some lakes, the shelter of the water area by the FPV facility can inhibit algae growth, thereby improving the aquatic environment and the water quality (Sharma et al. 2015). FPV facilities partially or fully covering the water surface will reduce water evaporation. Nevertheless, the FPV systems are planned on water surfaces with rich biodiversity, and the spacing needs to be considered so that sunlight can penetrate the water layer to reduce possible potential impacts, such as ensuring the dissolved oxygen content in the water. Currently, these studies have not revealed the mechanism of PV impact on water bodies. The main mechanism of the impact of PV arrays on the ecological environment is to disrupt the original radiant energy balance of the installation area, and then act on factors such as temperature, wind speed, turbulence, and precipitation (Armstrong et al. 2014). Given that ecosystem processes have a regulating effect on climate (Hu et al. 2016), it is important to understand the impact of PV power plants on the near-surface climate.

At present, the impact of PV power plants on the near-surface climate is mainly evaluated by modifying the albedo of the underlying surface where the PV array is located. The change in albedo is closely related to the PV array deployment areas. In terms of regional climate impact, Millstein et al. (Millstein and Menon 2011) studied the climate impact of large-scale deployment of PV arrays across the USA. The deployment of PV arrays in cities increases the albedo and reduces the regional temperature; but the deployment of arrays in the desert reduces the albedo and causes the temperature to rise. Among them, the kernel of the change in the deployment area of PV panels is the change in the background albedo, which indicates that the change in albedo depends on the background albedo in the deployment area. In this regard, Nguyen et al. (2017) used the Conformal Cubic Atmospheric Model (CCAM) to simulate and analyze the potential impact of large-scale PV arrays on the Australian climate. A large-scale PV array was controlled and simulated, and 80 sensitivity experiments were designed. The scale of each array was about 250,000 km^2^. The array direction and its location in Australia are arbitrary. The surface albedo of 20 PV arrays was set to four fixed values of 0.05, 0.25, 0.50, and 0.75. The albedo of 0.05 was lower than the background albedo of any location in Australia, 0.5 and 0.75 were higher than the background albedo, and the albedo of 0.25 depended on the geographic location of the array. The research results show that the impact of the solar PV field on its surrounding areas depends on the albedo change (the difference between the array albedo and the original background albedo), size, and direction of the array. Enlarging the size of the PV array will increase the albedo more than the original background albedo, which will have a greater impact on the ambient temperature and rainfall. In terms of urban climate impact, more research has shown that the impact of urban PV development is inducing a heat island effect. However, the impact of the heat island effect depends on the albedo change. Many studies have shown that the deployment the PV arrays on a building surface can reduce the surface temperature, reduce energy consumption, and alleviate the heat island effect. It can be seen that the impact of the PV power plants on air temperature due to the change in albedo is not uniform. Therefore, we established a model to explain this phenomenon, and provide data support and scientific basis for the sustainable development of solar PV.

## Site and method

### Site description

The study area is situated in Yangzhong City, Jiangsu Province, which is located in the middle of the northern subtropical monsoon climate zone, and has a mild climate, abundant rainfall, and the same season of rain and heat. In 2019 (January–December), the average temperature was 17.1 °C in Yangzhong City, the annual precipitation was 791.8 mm, and the annual accumulated sunshine time was 1792.2 h. The observation tower (32°18′9.00″ N, 119°47′33.45″ E, elevation 2 m) is located in the 10 MW FPV demonstration base in Tongwei Huantai, Yangzhong City. The first phase of the fishery complementary PV demonstration base is composed of four 2.3–3.6-ha ponds 2.5–3 m deep, separated by a path approximately 3 m wide. The center of the pond houses a PV power plant. The PV panels are fixed on the brackets installed on reinforced concrete columns spaced 6 m apart. The specification of each PV panel is 1.64 m × 0.99 m, and the tilt is 34.6°. Measured on June 4, 2020, the distance between the front edge and the rear edge of the PV panel was 1.6 m and 2.9 m, respectively.

**Fig. 1:**
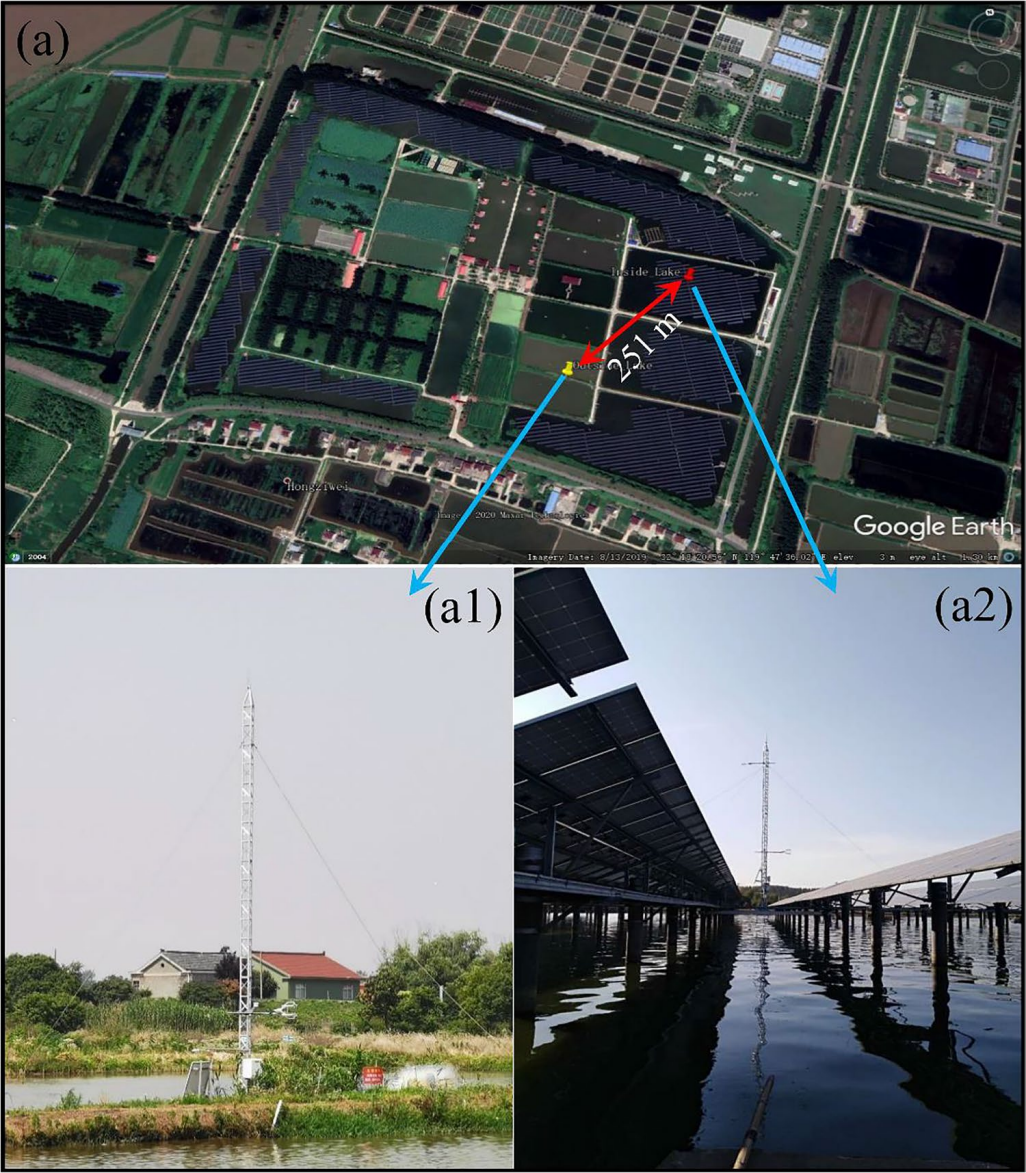
Study area of FPV power plant. **a** The FPV power plant is located in Yangzhong City, Jiangsu Province. The red pin represents the position of the meteorological tower inside the photovoltaic (PV) power plant. The yellow pin represents the position of the meteorological tower outside the PV power plant. (a1) is the meteorological tower outside FPV. (a2) is the meteorological tower inside FPV

The four-component net radiation sensor (CNR4, Kipp & Zonen) was mounted at an elevation of 10 m with an observation angle of 125°, and observation radius at an elevation of 10 m of 19.2 m. The water temperature was measured at three layers, 0.05 m, 0.75 m, and 1.5 m deep. The water temperature measurement line was attached to the buoy and rose or fell as the water level changed to ensure that the position of each probe from the water surface was basically unchanged. The eddy related system (IRGASON-IC-BB, Campbell Scientific) was installed at an elevation of 4.5 m, which was 2 m higher than the highest point of the PV panel to avoid the impact of the PV panel on the horizontal wind uplift. The center coordinates of the control observation tower outside the PV array were 32°18′4.60″ N and 119°47′25.30″ E, and the observation tower base was 2 m away from the edge of the pond to ensure that the observation range of the four-component radiometer was on the water surface to avoid any influence from the pond shore path. The installation height of the four-component radiometer was 2 m. The distance of the meteorological towers inside and outside the PV power plant was 251 m, and the specific relative positions are shown in Fig. 1.

Data collection of Eddy Covariance system (IRGASON-IC-BB, Campbell Scientific, Inc.) started on November 15, 2019. The high-frequency data (10 Hz) were stored in a CR3000 data logger (Campbell Inc., USA) and half-hourly mean flux were calculated online using the EddyPro software. The ECS was not maintained in time because the impact of 2019 novel coronavirus and the solar power supply system inside the PV has failed. The observation system stopped working after January 9, 2020, and normal measurement was performed only after maintenance on June 4, 2020. The daily data span was from June 2020 to October 2020 in this study.

### Method

The model established in this study is suitable for the calculation of energy changes after installing solar PV panels on any underlying surface, but for the convenience of presentation, only the lake surface is taken as an example. When solar PV panels are not installed, the energy received on the surface of the lake $Q_{lake}$ can be expressed by Eq. (1),

1 $$Q_{lake} = Q_{solar} \times (1 - \alpha_{lake} )$$

where $Q_{solar}$ is the solar radiation reaching the lake surface and $\alpha_{lake}$ is the lake surface albedo. After the solar PV panels are deployed, the energy received on the lake surface is recorded as $Q^{\prime}_{lake}$, which can be calculated by Eq. (2), where $\varepsilon$ is the solar conversion efficiency.

2 $$Q^{\prime}_{lake} = Q_{solar} \times [1 - (\alpha_{solar} + \varepsilon )]$$

Considering the gap after the PV panel is deployed, the total albedo after the PV panel and the lake surface are mixed at this time is $\alpha_{sum}$, and is calculated by Eq. (3), where $\varphi$ is the percentage of PV panel deployment.

3 $$\alpha_{sum} = (\alpha_{solar} + \varepsilon ) \times \varphi + \alpha_{lake} \times (1 - \varphi )$$

After PV installation, the energy $Q^{\prime\prime}_{lake}$ received by the lake surface considering the gap can be expressed by Eq. (4); $\alpha_{solar}$ is the PV panel albedo.

4 $$Q^{\prime\prime}_{lake} = Q_{solar} \times (1 - \alpha_{sum} )$$

The energy change $Q_{s}$ of the lake surface before and after the PV panel is installed can be obtained by Eq. (5):

5 $$Q_{s} = Q^{\prime\prime}_{lake} - Q_{lake} = Q_{solar} \times \varphi \times (\alpha_{lake} - \alpha_{solar} - \varepsilon )$$

It can be seen from Eq. (5) that $Q_{s} \propto [\alpha_{lake} - (\alpha_{solar} + \varepsilon )]$, where $\alpha_{lake}$ is the surface properties of the lake and $\alpha_{solar} + \varepsilon$ describes the properties of PV panels. $Q_{s}$ has three situations as in Eq. (6):

6 $$Q_{s} \left\{ {\begin{array}{*{20}c} { > 0} & {\alpha_{lake} > \alpha_{solar} + \varepsilon } \\ { = 0} & {\alpha_{lake} = \alpha_{solar} + \varepsilon } \\ { < 0} & {\alpha_{lake} < \alpha_{solar} + \varepsilon } \\ \end{array} } \right.$$

Therefore, the energy change after the PV panel is deployed mainly depends on the albedo of the underlying surface, the PV panel albedo, and solar conversion efficiency. In this study, the optimal value of the PV deployment ratio was 0.75 according to preliminary testing (Li et al. 2020), and solar conversion efficiency was 0.15 (Chang et al. 2020).

The albedo is the ratio of upward shortwave radiation divided by downward shortwave radiation (Li et al. 2022), calculated by Eq. (7).

7 $$\alpha = \frac{USR}{{DSR}}$$

The average value of $Q_{s}$ is calculated by the mean coefficient of determination (*R*^2^) of the fitting between $Q_{s}$ and counterpart environmental factors from June to October.

## Results and discussion

### Albedo

**Fig. 2:**
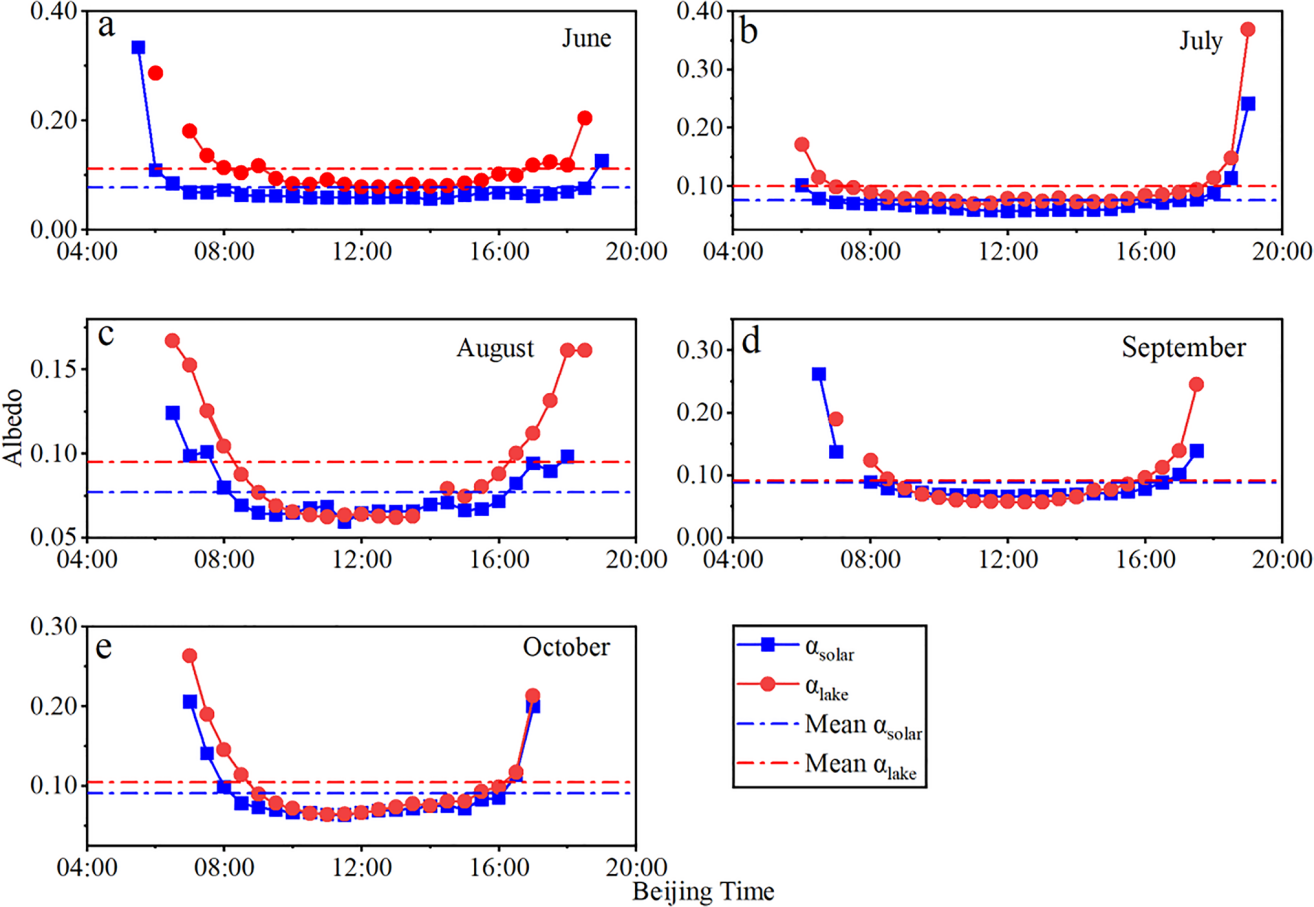
Diurnal characteristics of albedo inside and outside the fishery complementary PV power plant from June 2020 to October 2020 ((**a**) June; (**b**) July; (**c**) August; (**d**) September; (**e**) October)

Albedo determines the surface radiation balance and affects the climate (Argaman et al. 2012). The albedo of FPV deployed on the water surface is shown in Fig. 2. In addition, the $\alpha_{solar}$ and $\alpha_{lake}$ have the same meanings as Eqs. (1)–(6) in Sect. 2.2. Overall, the albedo presented a “U”-shaped change behavior. The average albedo of the free water surface and PV panel was 0.101 and 0.082, respectively. After the PV panels were installed, the albedo of the lake surface was reduced compared to the free water surface. According to Eq. (6), the free water surface can be turned into a heat source only by arranging PV panels in the lake regardless of solar conversion efficiency. The average albedo of the free water surface from June to October in 2020 was 0.112, 0.100, 0.095, 0.092, and 0.105, respectively, while with PV panel deployment, it was 0.078, 0.076, 0.077, 0.088, and 0.091, respectively. The average reduction in albedo caused by PV deployment was 18.65% during the study time span. The research results of Liu et al. (2018) on the Singapore floating PV plant showed that the albedo was between 0.05 and 0.07 and less than 0.082, mainly because the tilt of Liu’s PV panel was between 7 and 15°, while the tilt of the PV panel in this study was 34.6°, which is one of the factors that affect the albedo. However, the deployment of PV panels did not change the daily variation in albedo characteristics, but it decreased in value, with an average decrease of 18.65%. According to Eq. (6), it can be seen that without considering solar conversion efficiency, only the deployment of PV panels makes the lake surface a heat source, that is, $Q_{s} > 0$.

According to Eq. (6), when $Q_{s}$ is equal to 0, the solar conversion efficiency can be calculated as 1.88%. The current solar conversion efficiency with polysilicon materials is approximately 15%, which is far greater than 1.88%. That is, the lake surface after installing PV panels is converted from a heat source to a heat sink. Research by Chang et al. (2018; 2020) shows that PV power plants are also an energy sink. The change in energy is dispersed for PV panel deployment with different underlying surfaces but is dominated by the difference of the albedo between natural underlying surface and comprehensive underlying surface. Moreover, solar conversion efficiency is a major factor for the change in energy, although it is mainly related to the PV panel material (Fouad et al. 2017). As the albedo is related to the change in energy, we investigated the energy flux pattern in this study.

### Energy flux

**Fig. 3:**
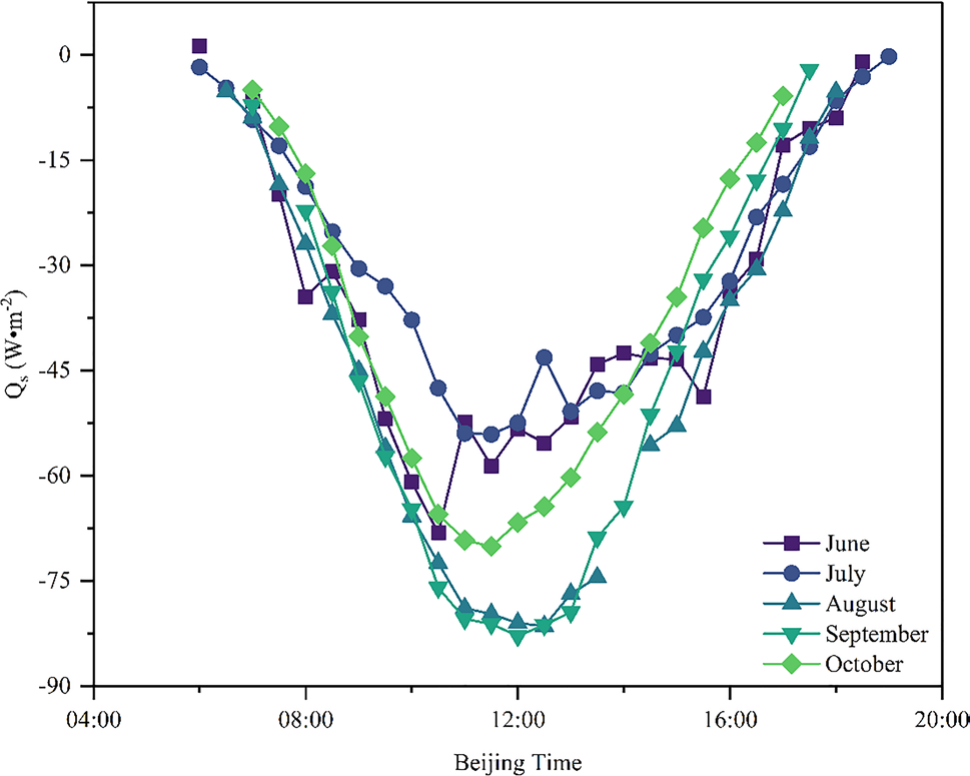
Energy changes on the lake surface after installing PV panels

The energy flux on the lake surface is shown in Fig. 3. The change in lake surface energy after installing PV panels was calculated by Eq. (5), where $Q_{solar}$ is the total surface radiation, the solar conversion efficiency $\varepsilon$ is 0.15, and the percentage of PV panels in the lake area $\varphi$ is 0.75. It can be seen from Fig. 3 that the overall appearance is a “V”-shaped change. On a daily scale, the power absorbed by PV panels gradually increased from 6:00 to 12:00, and the average power absorption change was 7.07 W·m^−2^ per hour, reaching the maximum at 12:00, with an average of 67.23 W·m^−2^. From 12:00 to 19:00, the power absorbed by the PV panels gradually decreased, and the average power absorption change was 5.57 W·m^−2^ per hour. On a seasonal scale, the trend of power absorbed by PV panels from June to October was basically the same, but the peak at 12:00 each month was different. The average power absorbed at 12:00 in August and September was 81.90 W·m^−2^, but was relatively small, with an average of 52.85 W·m^−2^ in June and July.

**Fig. 4:**
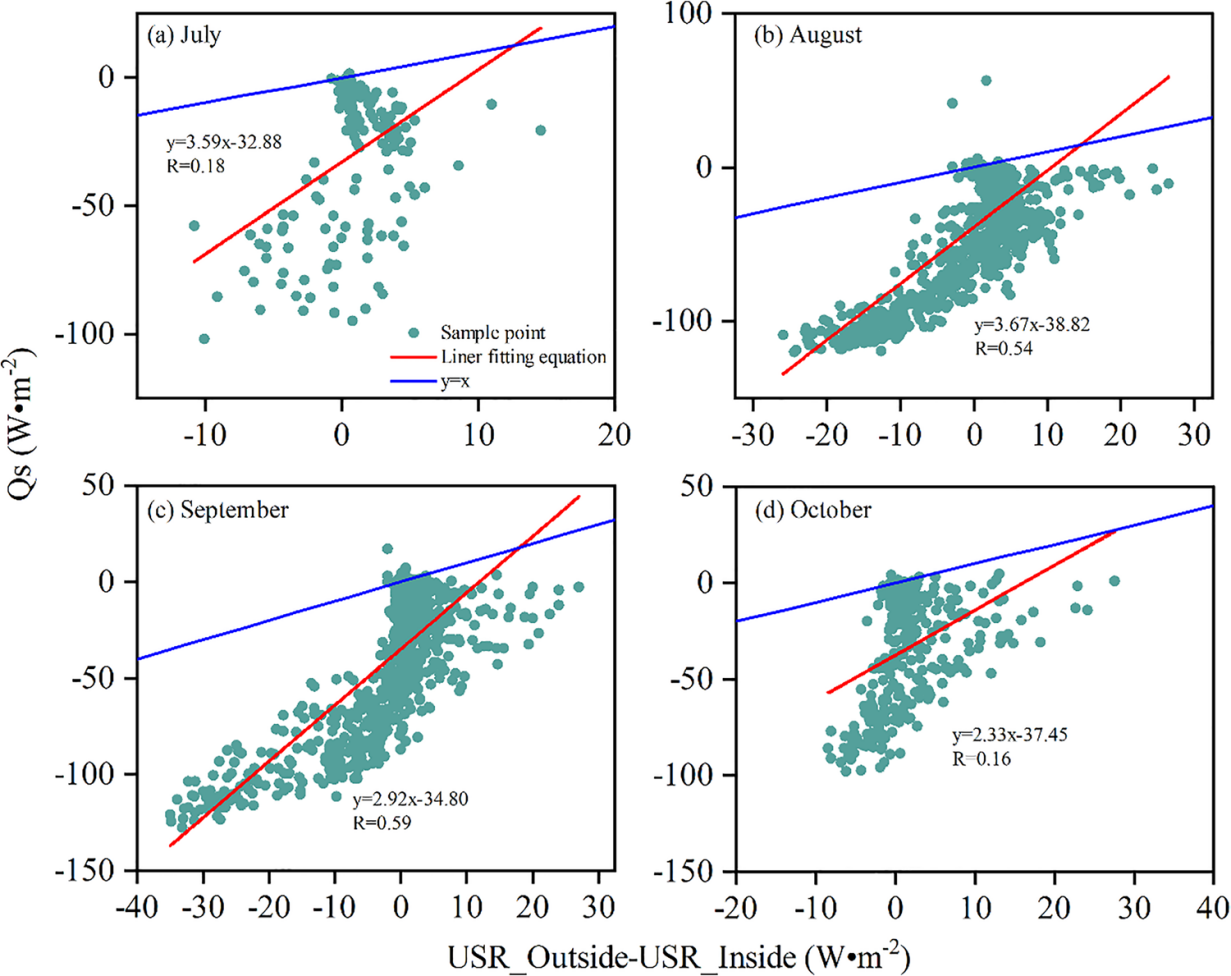
Comparisons between *Q*_s_ and the difference of USR_Outside and USR_Inside from July to October 2020

The *Q*_s_ is the energy change on the lake after the deployment of PV panels by the model calculation. The difference in upward surface shortwave radiation (USR, USR_Outside-USR_Inside) was calculated from the observations of the two meteorological towers inside and outside the FPV power plant (Fig. 4). The *Q*_s_ is equal to the difference in USR in two sites in theory; however, the *Q*_s_ was higher than the observation of USR at the two sites. When the range of difference in USR was between − 5 W·and 5 W·m^−2^ from July to October, the model performance was good. For the period of robust power generation, the difference in energy change at two sites was not captured by the model because the solar conversion efficiency was constant in this study. This parameter depends on the weather (sunny, cloudy, and rainy) and solar radiation. In addition, the area shielded by the FPV panel can also impact the model. We took into consideration into the model of energy change after deployment of FPV panels on the lake. Therefore, we considered those impact factors in the model to improve its performance and accuracy.

### Environmental factors

**Fig. 5:**
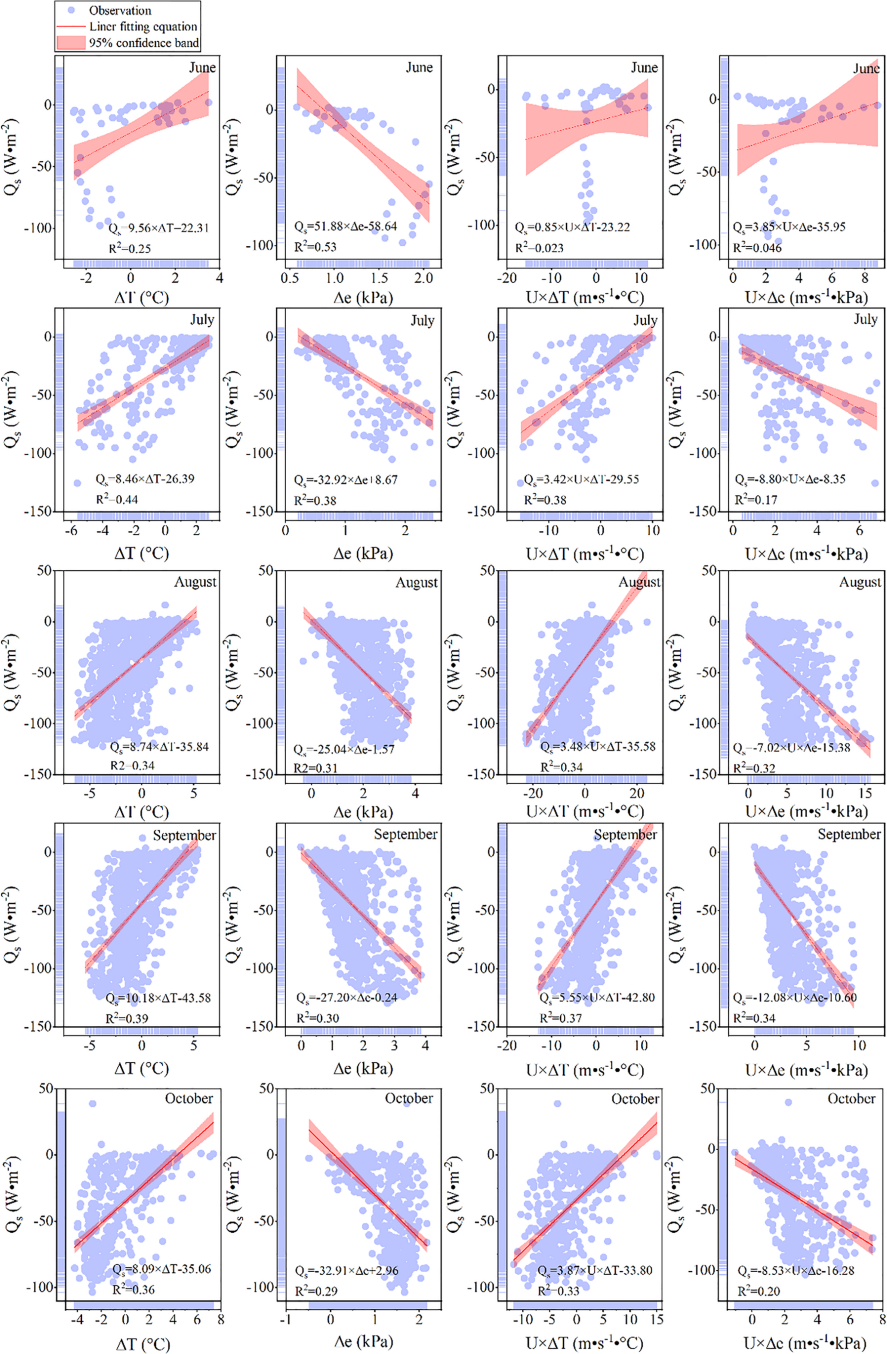
Driving force of changes in lake surface energy inside the fishery complementary PV power plant from June 2020 to October 2020. (a1–a4) Changes in lake surface energy as a function of ∆*T* (water–atmosphere temperature difference, °C), ∆*e* (water–air vapor pressure deficit, kPa), the product between *U* (wind speed, m·s^−1^) and ∆*T*, and the product between *U* and ∆*e* in June. (b1–b4) Same as a1–a4 but in July. (c1–c4) Same as a1–a4, but in August. (d1–d4) Same as a1–a4, but in September. (e1–e4) Same as a1–a4, but in October 2020

The relationship between the changes in lake surface energy and environmental factors caused by the deployment of PV panels is shown in Fig. 5. In general, except for in June, *Q*_s_ was positively correlated with the water–atmosphere temperature difference (∆*T*, °C), the product between wind speed (*U*, m·s^−1^) and ∆*T*, and negatively correlated with water–air vapor pressure deficit (∆*e*, kPa), the product between U and ∆e.

The average results of ∆*T* and ∆*e*, the product between *U* and ∆*T*, and the product of *U* and ∆*e* for *Q*_s_ were 35.6%, 36.2%, 28.9%, and 21.5%, respectively. It can be seen that the explanatory effect of ∆*e* for *Q*_s_ is higher than that for other environmental factors.

**Fig. 6:**
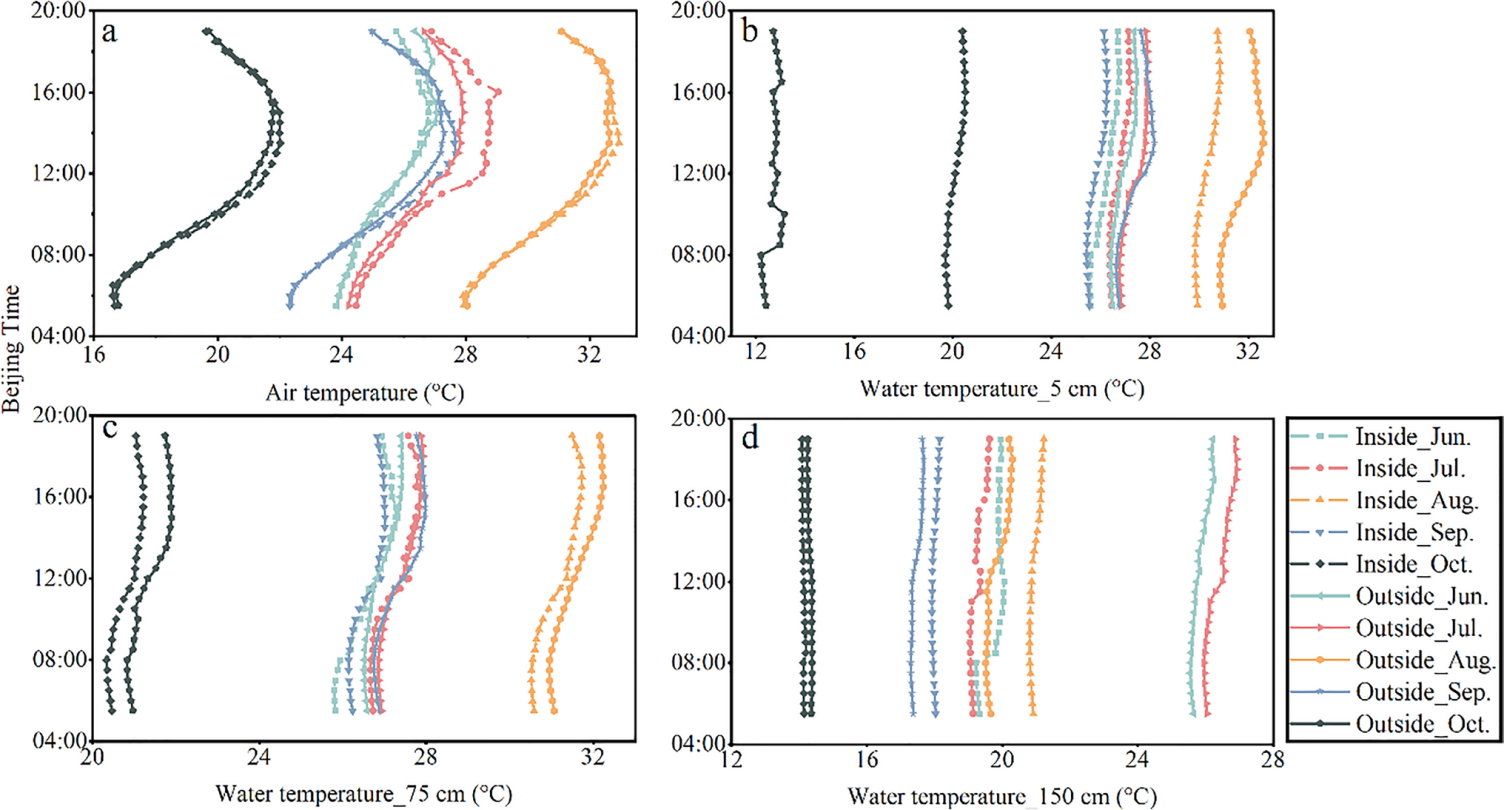
Comparison of air temperature and water temperature inside and outside the PV power plant from June to October 2020 ((**a**) 2-m air temperature; (**b**) 0.5-m water temperature; (**c**) 0.75-m water temperature; (**d**) 1.5-m water temperature. The solid line represents the temperature outside the PV power plant, the dash-dot line represents the temperature inside the PV power plant)

In addition, the driving force of changes in lake surface energy is clear except in how the lake surface energy affects the ambient temperature. The air temperature and water temperature are explored to reveal the energy partitioning in the lake. The changes in air temperature and water temperature of the three layers in two sites are shown in Fig. 6. It can be seen that the average air temperature of 2 m in the PV site from June to October was 0.16 °C higher than that outside the plant. On the daily scale, the temperature difference between inside and outside PV increased with the amplification of solar radiation, and the temperature difference in September was as high as 0.49 °C. Studies have also shown that onshore PV power plants have a significant heating effect on ambient air at an elevation of 2 m during the day (Barron-Gafford et al. 2016, Broadbent et al. 2019, Chang et al. 2018, Yang et al. 2017, Yang et al. 2015). However, Liu et al. (2018) studied eight different floating PV plans in Singapore’s Tengeh Reservoir in which the water surface temperature was 1–3 °C lower than onshore, which may be related to the tilt of PV panels, water area, and PV power plant. Factors such as location and scale are related, and the specific reasons need further study. The temperature inside and outside the station in August was higher than that in other months, and the average temperature inside and outside the plant was 31.22 °C and 31.13 °C, respectively. The temperature inside and outside the plant in October was lower than that in other months, and the average temperature inside and outside the plant was 20.06 °C and 19.96 °C, respectively.

In August, the water temperature 0.5 m outside the plant was higher than that inside the plant. The average water temperature inside and outside the plant was 26.22 °C and 26.94 °C. In October, the water temperature 0.5 m inside the plant was higher than that outside the plant, and the average water temperature inside and outside the plant was 20.12. °C and 12.74 °C. It can be seen that the impact of PV panel deployment on the lake water temperature was not constant. In August, the water temperature 0.5 m inside the plant was lower due to the shading effect of the PV panels. In October, the temperature 0.5 m inside the plant was caused by the heating effect of the PV panels. The water temperature was higher than that outside the plant. From July to September, the shadowing effect of PV panels on the 0.5-m water temperature was greater than the warming effect; that is, the water temperature inside the plant was lower than the water temperature outside the plant.

The 0.75-m water temperature of the lake from June to October showed that the water temperature outside the plant was higher than that inside the plant. The average water temperature inside and outside the plant in August was 26.63 °C and 26.94 °C; the average water temperature inside and outside the plant in October was 20.83 °C and 21.41 °C. The 0.75-m water temperature difference between inside and outside the plant was significantly smaller than the 0.5-m water temperature difference. The water temperature difference in August and October decreased by 56.94% and 92.14%, respectively, indicating that PV panels have a greater impact on the surface water temperature.

The 1.5-m water temperature of the lake was not the highest in August, because the change in water level makes the probe touch the silt at the bottom of the lake. The average water temperature inside and outside the plant was 20.97 °C and 19.85 °C, respectively. The water temperature outside the plant from June to July was significantly higher than that inside the plant, and the water temperature outside the plant (14.34 °C) in October was also slightly higher than that inside the plant (14.14 °C). Therefore, when the 1.5-m water temperature was higher than 24 °C, the shading effect of the PV panel is obvious. Chang et al. (2018) also found that PV power plants in desert areas have a shading effect, which reduces the monthly average surface temperature by 1.8 to 8.2 °C. However, the annual average temperature of PV panels is 3.8 °C higher than the 2-m air temperature. Therefore, a PV power plant heating effect also exists. However, the shielding effect and heating effect formed by PV power plants on diverse underlying surfaces have different impacts on the ambient temperature. When the 0.75-m water temperature is lower than 24 °C and the 1.5-m water temperature is lower than 16 °C, the depth of the heating effect of the PV panel on the water temperature is limited, and this effect cannot reach the water layer of 0.75 to 1.5 m.

## Conclusions

This research presents a simple model to study the environmental impact of a PV power plant on the water surface underneath. The following conclusions are derived from the model analysis and investigation:

1. The water surface albedo in PV panel deployment areas (0.082) was decreased by 18.8% relative to the albedo of the free water surface (0.101) during the observational period.
2. The lake became a heat sink after installing the PV array at a daily scale, and the average power absorption change was 7.07 W·m^−2^ per hour.
3. The water energy changes were explained by 36.2% through the water–air vapor pressure deficit.
4. The PV array had a heating effect on the ambient temperature, but it was limited by water depth.

## Data Availability

The datasets used during the current study are available from the corresponding author on reasonable request.
